# Invited Review: Decoding the pathophysiological mechanisms that underlie RNA dysregulation in neurodegenerative disorders: a review of the current state of the art

**DOI:** 10.1111/nan.12187

**Published:** 2015-01-29

**Authors:** M J Walsh, J Cooper-Knock, J E Dodd, M J Stopford, S R Mihaylov, J Kirby, P J Shaw, G M Hautbergue

**Affiliations:** Sheffield Institute for Translational Neuroscience (SITraN), Department of Neuroscience, University of SheffieldSheffield, UK

**Keywords:** altered gene expression, neurodegeneration, protein loss-of-function, RNA/protein toxic gain-of-function, RNA-mediated diseases

## Abstract

Altered RNA metabolism is a key pathophysiological component causing several neurodegenerative diseases. Genetic mutations causing neurodegeneration occur in coding and noncoding regions of seemingly unrelated genes whose products do not always contribute to the gene expression process. Several pathogenic mechanisms may coexist within a single neuronal cell, including RNA/protein toxic gain-of-function and/or protein loss-of-function. Genetic mutations that cause neurodegenerative disorders disrupt healthy gene expression at diverse levels, from chromatin remodelling, transcription, splicing, through to axonal transport and repeat-associated non-ATG (RAN) translation. We address neurodegeneration in repeat expansion disorders [Huntington's disease, spinocerebellar ataxias, C9ORF72-related amyotrophic lateral sclerosis (ALS)] and in diseases caused by deletions or point mutations (spinal muscular atrophy, most subtypes of familial ALS). Some neurodegenerative disorders exhibit broad dysregulation of gene expression with the synthesis of hundreds to thousands of abnormal messenger RNA (mRNA) molecules. However, the number and identity of aberrant mRNAs that are translated into proteins – and how these lead to neurodegeneration – remain unknown. The field of RNA biology research faces the challenge of identifying pathophysiological events of dysregulated gene expression. In conclusion, we discuss current research limitations and future directions to improve our characterization of pathological mechanisms that trigger disease onset and progression.

## Introduction

RNA-mediated neurodegeneration is implicated in the causes of Huntington's disease (HD), spinocerebellar ataxias (SCAs), spinal muscular atrophy (SMA) and major subtypes of amyotrophic lateral sclerosis (ALS). In contrast, the current neuropathological classification of Parkinson's disease (PD), Alzheimer's disease (AD) and prion disease relates to the abnormal accumulation of misfolded and aggregated proteins in the brain (synucleinopathies, tauopathies and prion protein accumulation, respectively).

HD is a fatal disease initiated by selective death of neurons in the striatum before neurodegeneration spreads to other cerebral regions. Startlingly, some *post mortem* HD brains may have lost up to 25% of their weight. HD usually develops in patients aged 35–45 years, although onset can occur from childhood to old age. Caucasian populations have a high and likely underestimated prevalence (5–7 cases in 100 000 individuals). The disease progressively affects movement (chorea, particularly impaired swallowing/speech), behaviour and cognitive functions. Neuropsychiatric problems worsen over time and ultimately lead to dementia [1]. HD is caused by an autosomal-dominant glutamine-encoding CAG trinucleotide expansion in the polymorphic exon 1 of the huntingtin gene (*HTT*). Unaffected individuals normally carry 11–34 CAG repeats while HD patients have >36–250 CAG repeats [2,3].

The SCAs form a large heterogeneous group of autosomal-dominant diseases with typical onset at 30–50 years old. They are caused by repeat expansions in both coding and noncoding regions of multiple genes. Repeat expansions are formed by CAG repeat sequences in most cases; however, some subtypes are characterized by the presence of trinucleotide (CTG), pentanucleotide (ATTCT, TGGAA) or hexanucleotide (GGCCTG) repeats. SCAs are slowly progressive diseases involving neurodegeneration of the cerebellum and spinal cord. They are associated with dysarthria, poor coordination of gait and fine movements, but with retention of cognitive function [4]. They often remain undiagnosed.

SMA is the second most common genetic cause of infant mortality after cystic fibrosis (approximately 1 in 10 000 newborns). Lower motor neurons in the anterior horns of the spinal cord progressively degenerate, leading to muscle atrophy, paralysis and often fatal respiratory failure [5]. Babies affected by aggressive forms of SMA (type 0 and I) never sit and have a very short life expectancy (<2 years). Intermediate SMA type II children sit but do not stand. Types III and IV SMA have impaired gait and mobility; however, their disease does not affect life expectancy. Mental abilities remain unaffected and may be higher than average. This autosomal-recessive neurodegenerative disease is due to homozygous disruption of the survival of motor neuron 1 (*SMN1*) gene [6] which results in reduced levels of the ubiquitous SMN protein. The greater the reduction in SMN level, the greater severity of disease.

ALS is characterized by relentless degeneration of upper and lower motor neurons, which leads to progressive paralysis and death usually within 3–5 years from symptom onset. ALS is generally an adult disease with age of onset peaking at around 55–60 years of age. It is the most common form of motor neurodegenerative disease and has a prevalence of 6–10 cases per 100 000 individuals, with a lifetime risk of 1 in 400 [7]. Approximately 5% of ALS cases are inherited, usually in an autosomal dominant manner (familial ALS – fALS) while the majority of cases, approximately 95%, occur sporadically (sALS) [8]. ALS is a multifactorial disease in which mutations in multiple genes cause a direct disruption of mRNA metabolism 9–11.

Here, we review the molecular mechanisms by which genetic mutations can alter normal gene expression in neurodegenerative diseases. We also discuss research limitations and future strategies to better understand the functional cellular consequences of widespread RNA dysregulation.

## Eukaryotic expression of genes

Eukaryotic gene expression is tightly regulated and integrates activating and repressive mechanisms [12], directionality [13], RNA surveillance [14] and regulated protein degradation [15]. Normal eukaryotic gene expression depends on a very large number of protein-coding mRNAs, protein factors and noncoding RNAs (ribosomal, transfer, small nuclear RNA and regulatory micro-RNAs). RNA molecules associate with RNA-binding proteins to form ribonucleoprotein particles (RNPs). The composition of RNPs dictates the function and fate of the RNA molecules [16]. Most eukaryotic genomes encode hundreds of RNA-binding proteins with diverse biological activities [17]. Mutations that disrupt RNPs are prone to cause disease, whether they affect a particular protein or RNA molecule. In particular, several neurological disorders are associated with transcription and pre-mRNA splicing alterations [18,19], as well as with dysregulation of protein synthesis [20].

**Figure 1:**
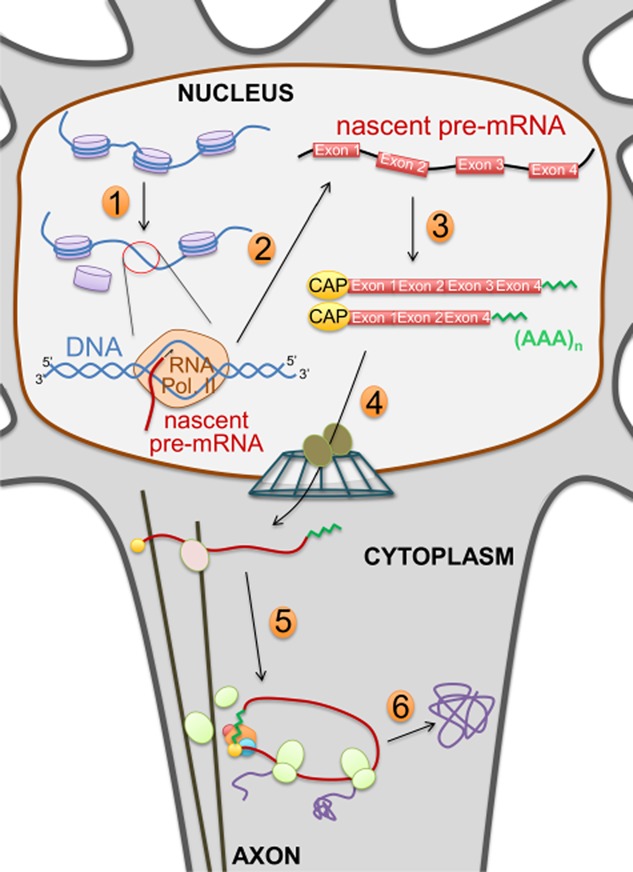
Neuronal expression of protein-coding genes. Diagram highlighting mRNA biogenesis and processing, nuclear export, axonal transport and mRNA translation. (1) Chromatin remodelling; (2) RNA polymerase II (RNA Pol. II) dependent transcription; (3) co-transcriptional processing: 5′-end capping, splicing/alternative spicing, 3′-end cleavage and poly-adenylation; (4) nuclear export of mRNAs; (5) axonal transport of mRNAs; and (6) translation of mRNAs for the biosynthesis of proteins.

Biogenesis/processing of mRNA is orchestrated in separate, but extensively coupled steps within the nucleus [21], including transcription, mRNA processing events (capping, splicing, cleavage/poly-adenylation) and nuclear export [22]. RNA-polymerase II transcribes pre-mRNAs from the genomic DNA [23]. Nascent mRNA transcripts are co-transcriptionally capped at their 5′-end [24]. The spliceosome removes introns and stitches together coding exons in a process called splicing [25]. Alternative splicing allows differential linking of various exons, increasing the repertoire of encoded proteins from a single gene. Finally, processing mRNAs are cleaved and poly-adenylated at the 3′-end [26,27]. Mature mRNAs that have been processed are licensed for nuclear export 28–30. Cytoplasmic mRNAs are circularized before being translated into proteins by the ribosome [31,32]; however, they can also be transported through the axon of neuronal cells [33] to allow localized translation of proteins within the axonal compartment. Figure 1 illustrates the mechanistic steps involved in the neuronal expression of protein-coding genes.

## HD

The *HTT* gene encodes a very large protein huntingtin (HTT) of over 3000 amino acids (approximately 350 kDa) which is essential for embryonic development and brain function [34]. The domain structure of HTT does not resemble any known proteins and its precise molecular function still remains unclear.

**Figure 2:**
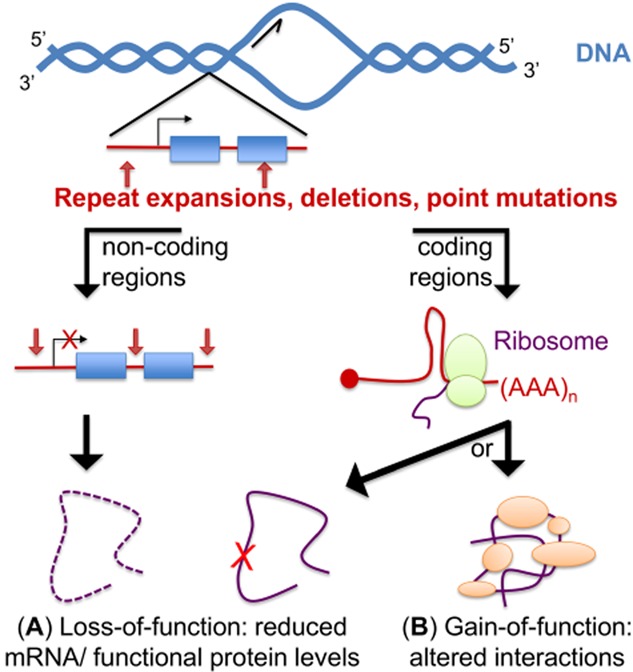
Mechanisms conferring protein loss and toxic gain-of-function effects. The diagram illustrates pathogenic mutations (repeat expansions, deletions, point mutations) that may occur either in noncoding or coding regions of the genome (left and right sides, respectively). (A) Protein loss-of-function. Haploinsufficiency can occur when the level of a particular mRNA is down-regulated due to mutations in noncoding regions of genes such as in promoters/introns, or if the promoter is subjected to histone/DNA modifications (transcriptional repression), but also if mutations in 5′ or 3′ untranslated regions (UTRs) decrease mRNA stability. Protein loss-of-function can also occur when mutations in coding regions alter directly the activity of the mutated protein (misfolding, alteration of the active site). (B) Protein toxic gain-of-functions are caused by mutations in coding regions that either promote abnormal interactions, increase the interaction of the mutated protein with its natural binders and/or promote misfolding/aggregation.

**Figure 3:**
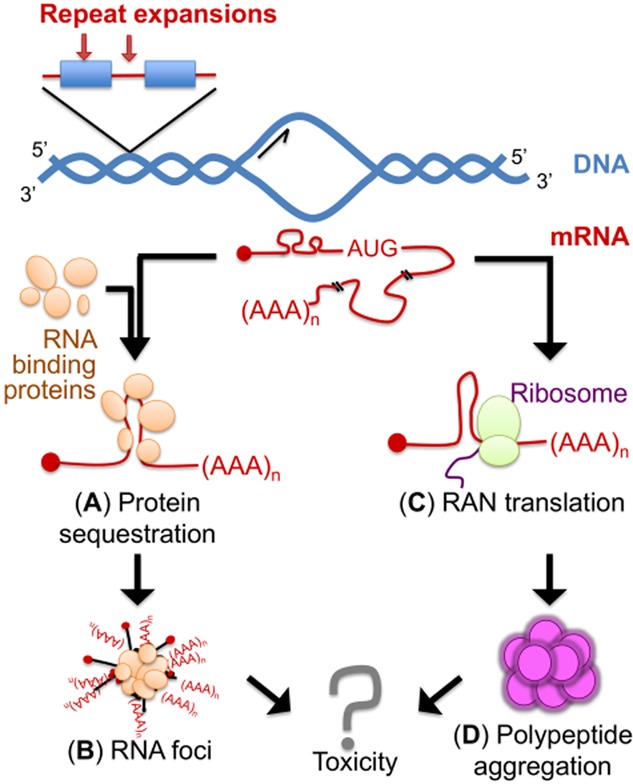
RNA toxic gain-of-function mechanisms. (A) Protein sequestration of RNA-binding proteins that avidly interact with the repeat expanded pre-mRNA/mRNA. (B) Formation of RNA foci. (C) Repeat-associated non-ATG (RNA) translation. (D) RAN translation leads to the formation of repeat-peptide proteins that usually aggregate.

Poly-glutamine (poly-Q) amino-terminal truncations of HTT, generated through aberrant splicing of *HTT* in HD [35], inappropriately accumulate within the nucleus through altered interactions with the nuclear pore protein translocated promoter region (Tpr) [36], and form ubiquitinated neuronal intranuclear inclusions in human [37] and mouse [38] HD brains. α-synuclein, a component of Lewy bodies in PD brains, was found in HTT inclusions and independently in cytoplasmic filaments in human and mice HD neurons. Interestingly, the number of HTT inclusions is dependent on α-synuclein expression levels [39], and formation of inclusion bodies was shown to be associated with improved survival rather than death upon live cell imaging [40]. HD pathophysiology is complex, and there are several pathophysiological mechanisms that lead to broadly dysregulated gene expression [41]. Approximately 200 mRNAs are dysregulated in HD brains, and the level of dysregulation correlates with disease severity in the affected brain regions 42–44. Poly-Q expansions trigger both HTT protein loss-of-function and toxic gain-of-function effects [45]. Figure 2 generally illustrates these mechanisms. In addition, the CAG expansion may also contribute to HD pathogenesis via RNA toxic gain-of-function through RNA foci formation and/or partial sequestration of the muscleblind (MBNL1) splicing factor [46] and nucleolin (NCL) [47]. Panels A and B of Figure 3 depict in general these paradigms/mechanisms. NCL sequestration leads to down-regulation of rRNA transcription and nucleolar stress. The CAG-expanded RNA is furthermore thought to cause toxicity by altering miRNA biogenesis [48].

Loss of HTT protein function plays a critical role in HD. HTT is involved in various cellular functions [49], particularly in the nervous system. It protects against excitotoxicity [50] and apoptosis by promoting mitotic spindle formation during neurogenesis [51]. It regulates the axonal transport of vesicles, regulating in turn synaptic transmission [52]. It contributes to miRNA biogenesis [53]. Furthermore, HTT sequesters repressor element 1 silencing transcription (REST) in the cytoplasm of neurons, leading to the transcriptional activation of REST-repressed genes involved in neuronal differentiation and survival [54]. Loss of functional cytoplasmic HTT in HD leads to nuclear accumulation of REST and subsequent down-regulation of REST-regulated neuronal genes [54]. These include brain-derived neurotrophic factor (BDNF) which encodes a cortical pro-survival factor essential for striatal neuron survival [55] and several neuronal miRNAs [56] that regulate the plasticity of the neuronal transcriptome [57,58].

On the other hand, the poly-Q expansions in HTT also trigger several toxic gain-of-functions described below. HTT has the ability to interact with over 100 proteins involved in various cellular functions [49]. Poly-Q expansions either disrupt or cause abnormal HTT: protein interactions, thus affecting many cellular pathways [59,60].

1. Poly-Q HTT aberrantly interacts and may sequester several transcription factors (TFs) which disrupt transcription and widely affect gene expression. Several TFs have been found in nuclear HTT inclusions, including the general transcription factor TATA box binding protein (TBP) and co-transcriptional activators such as CREB-binding protein (CBP) and specificity protein 1 (SP1) [59,61] which contribute to the establishment of neuronal identity.
2. Poly-Q HTT directly binds to the promoter of peroxisome proliferator-activated receptor gamma coactivator 1-alpha (*PPARGC1A*), as well as its protein product PGC-1α, a transcriptional master co-regulator that regulates energy homeostasis, mitochondrial biogenesis and antioxidant defences. Reduced *PPARGC1A* transcription and disrupted PGC-1αfunction are likely to contribute to the mitochondrial dysfunction observed in HD [62], a key pathophysiological component in combination with increased oxidative stress and mitochondrial DNA damage [63]. Interestingly, oxidative stress promotes DNA triplet expansion in *HTT* [64], highlighting the potential pathophysiological role of DNA damage/repair during neurogenesis. In addition, rescuing PGC-1α function attenuates HD in mice, alleviating both oxidative stress and HTT aggregation proteotoxicity [65].
3. Poly-Q HTT also binds the acetyltransferase domains of the histone acetyltranferases (HATs) CBP and P/CAF. Post-translational modifications of histones control the accessibility of the chromatin to the transcriptional machineries. Alteration of chromatin remodelling in HD leads to widespread dysregulation of gene transcription in large genomic regions [43]. This occurs through reduction of histone H3/H4 acetylation [66], reduction of histone H3 phosphorylation at the PGC-1α promoter [67] and decreased interaction with the human polycomb-repressive complex 1-like (hPRC1L) E3 ubiquitin ligase complex that promotes mono-ubiquitination of histone H2A [68].
4. The expanded HTT RNA [48] and protein [53] have furthermore a direct role in the alteration of miRNA biogenesis.
5. Finally, CBP dysregulation also results in reduced acetylation of the rRNA-specific upstream binding factor 1 (UBF1) which dysregulates rRNA transcription and leads to nucleolar stress [69].

## SCAs

There are 36 SCA subtypes identified to date, with a combined prevalence of 5–7 cases per 100 000 people 70–72. Most SCA subtypes are caused by repeat expansion mutations that occur in over 20 known genes. SCAs can be sporadic, or have autosomal recessive or autosomal dominant inheritance dependent on the subtype. The most widely characterized genetic subset are the autosomal dominant cerebellar ataxias (ADCAs), which are subdivided into three broad types based on their clinical presentation [73].

Many of the ADCAs (SCA1-3, SCA6, SCA7, SCA17) are caused by the presence of expanded CAG repeats within exonic regions of genes. These repeats are reminiscent of those found in HD and have led to these SCAs being classified as poly-Q diseases [74]. Misfolded poly-Q-containing proteins or soluble glutamine-rich peptides (created by their cleavage) are thought to cause neuronal toxicity through toxic gain-of-functions, abnormal interactions and/or protein aggregation [75] (Figure 2). The SCA poly-Q proteins are proposed to disrupt the ubiquitin-proteasome system [76,77], alter calcium homeostasis [78] and dysregulate transcription 79–81.

Other SCA subtypes such as SCA10, SCA31 and SCA36 are caused by a variety of different noncoding repeat expansions, which have the potential to cause RNA toxicity important in disease pathogenesis [82] (Figure 3**A**–**C**). An ATTCT pentanucleotide repeat expansion in intron 9 of *ATXN10* causes SCA10 [83]. Affected individuals have between 800 and 4500 repeats. Intron 9 is spliced out of the *ATXN10* pre-mRNA, but the expanded AUUCU RNA is resistant to degradation and aggregates in nuclear and cytoplasmic foci of SCA10 cells and transgenic mouse brain. The expanded AUUCU RNA binds the splicing factor heterogeneous nuclear ribonucleoprotein K (hnRNPK), resulting in hnRNPK sequestration and loss of function. As a result, protein kinase C δ (PKCδ) accumulates in the mitochondria of SCA10 cells, leading to caspase-3 mediated apoptosis [84]. SCA31 is caused by a TGGAA pentameric repeat expansion located within the intron of both brain-expressed associated with NEDD4 (*BEAN*) and thymidine kinase 2 (*TK2*), genes that are transcribed in the opposite direction. Affected individuals have over 250 repeats. The expanded UGGAA RNA binds and sequesters the serine arginine-rich splicing factor (SRSF) SRSF1 and SRSF9 *in vitro* [85]. If these splicing factors are sequestered *in vivo*, pre-mRNA processing and stability are likely to be disrupted. SCA36 is intriguing because it exhibits similarities with C9ORF72-related ALS. SCA36 patients initially develop cerebellar ataxia and frontal lobe atrophy. However, symptoms typical of ALS follow, with upper and lower motor neuron involvement, including tongue atrophy, skeletal muscular atrophy and fasciculation [86,87]. SCA36 is caused by a GGCCTG hexanucleotide repeat within the first intron of nucleolar protein 56 (*NOP56*) [88], while C9ORF72-related ALS is caused by an expanded GGGGCC hexanucleotide repeat within intron 1 of *C9ORF72* [89,90]. C9ORF72-related ALS and SCA36 are linked not only because of motor neuron dysfunction, but also because both intronic XGGGCC repeat expansions (where X is G or T respectively) interact with SRSF2 and form RNA foci [88,^91^,92]. The expanded GGCCTG repeat also leads to reduced expression of the neighbouring *miR-1292* gene [88].

In some other SCAs, there are contributions from both protein gain-of-function and RNA toxicity. The expanded CAG repeat in SCA3 not only produces poly-Q proteins, but also produces expanded CAG repeat RNA that potentially binds and sequesters RNA-binding proteins. The dysregulated transcription and splicing could contribute to neurodegeneration [93,94]. In addition, SCA8 is caused by contribution from a CTG repeat expansion in ataxin (*ATXN*) *ATXN8OS* and a CAG repeat expansion in *ATXN8*, genes which are transcribed in opposite directions [95]. *ATXN8OS* is transcribed from the sense strand, and the transcript contains an untranslated CUG repeat, while the CAG repeat expansion within *ATXN8* on the antisense strand is translated into an almost pure poly-Q protein. The CUG repeat-containing RNA forms nuclear foci which sequesters the RNA processing factor MBNL1 [96]. MBNL1 sequestration has adverse effects on RNA splicing and other processing events, and is well studied in myotonic dystrophies 1 and 2. Additionally, the CAG repeats are translated into poly-Q proteins, and in a different frame into polyalanine proteins via unconventional repeat-associated non-ATG (RAN) translation in SCA8 mouse model and *post mortem* human central nervous system (CNS) tissue [97].

## SMA

SMA is caused by a drastic reduction of SMN protein levels. The chromosomal 5q13 SMA locus is a duplicated region carrying two inverted copies of almost identical *SMN* genes that encode the same 294-residue protein. The majority of SMA cases show homozygous deletions, rearrangements or large truncations of the telomeric *SMN1* gene copy. However, other cases are caused by short deletions/mutations in the splice sites of *SMN1* introns 6 and 7. The *SMN1* gene differs from the centromeric *SMN2* copy by a few silent base changes [6]. Exon-7 is correctly spliced in only 10–20% of *SMN2* transcripts, leaving the vast majority of SMN2 mRNAs as defective transcripts lacking exon-7 [6,98] which are eventually translated into unstable and inactive SMN protein [99,100]. Significantly, *SMN2* alterations are not associated with clinical pathology. Healthy motor neurons naturally express lower amounts of fully spliced *SMN2* mRNA which may account for the higher vulnerability of motor neurons to *SMN1* mRNA loss [101]. Because SMN protein levels are directly linked to disease severity, it is critical to identify the mechanisms that regulate inclusion/exclusion of *SMN2* exon-7 for the development of therapeutic approaches. As described below, the intricate regulation of exon-7 splicing involves binding of multiple RNA recognition motif (RRM) containing proteins such as SRSFs and hnRNPs that act directly on binding exon-7 and flanking introns as well as through direct protein : protein interactions.

**Figure 4:**
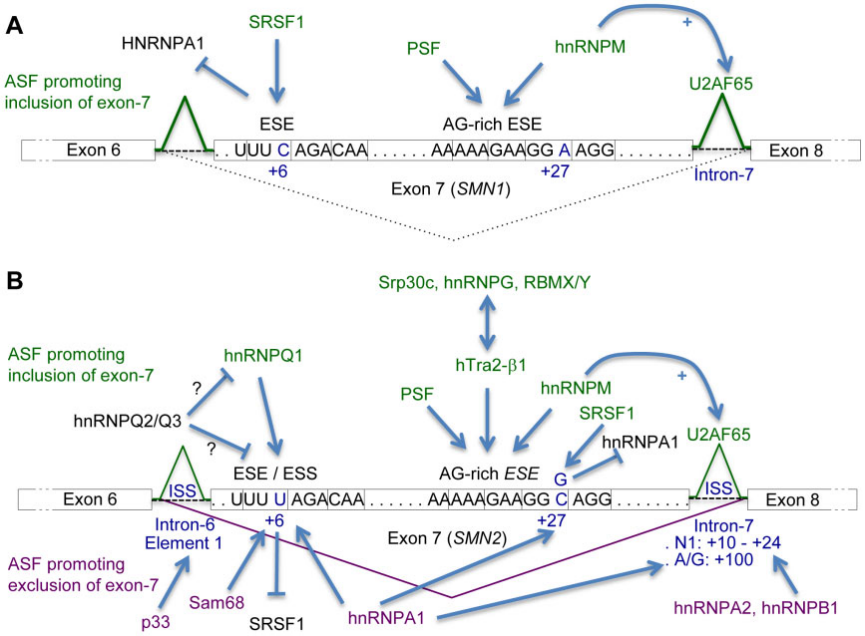
Model for regulation of exon-7 splicing in SMN1 and SMN2. Schematic representation of positive and negative effectors that regulate exon-7 splicing in *SMN1* (A) and *SMN2* (B) genes. The acronym ASF was used for alternative splicing factors. Exons 7 of SMN1 or SMN2 are represented in boxes that include the DNA sequences of the ESE/ESS motifs. ISS sequences are located in introns flanking exon 7 of *SMN2*. Arrows represent binding of ASF to the highlighted DNA elements or proteins. Factors that promote or inhibit the inclusion of exon-7 are respectively labelled above or below SMN exons/introns. T lines represent binding inhibition/inhibitory effect of ASF. (A) SRSF1 recognizes a +6 ESE sequence in *SMN1* exon-7 promoting inclusion of exon-7. A downstream AG-rich ESE in exon-7 promotes exon-7 inclusion through binding of PSF [102] and hnRNPM [103], which in turn stimulates the recruitment of the splicing factor U2AF65 to the flanking intron-7. (B) The ESE sequence altered by a C/T transition at position +6 in *SMN2* exon-7 was initially suggested to reduce exon-7 splicing because of a decreased interaction with SRSF1 [98,^104^,105]. However, the C/T transition also forms a composite ESS that promotes exon-7 skipping by interaction with the alternative splicing inhibitors hnRNPA1 [106,107] and Sam68 [108]. Furthermore, the activities of both hnRNPQ2 and Q3 antagonize the positive exon-7 splicing role of hnRNPQ1 bound to the +6 ESE [109]. Several base changes in *SMN2* introns 6 and 7 also promote *SMN2* exon-7 exclusion: (i) an ISS *Element 1* in intron-6 (−75 to −89) through binding of p33 [110]; (ii) an *ISS-N1* site located in intron-7 (+10 to +24) that provides binding sites for hnRNPA2 and B1 [111]; (iii) an ISS in intron-7 (A/G transition at position +100) that binds hnRNPA1 and inhibits splicing of exon-7 cooperatively with the binding of the same protein to the exon-7 ESS site [112]. In contrast, *SMN2* exon-7 inclusion is promoted via two ESE sites: (i) the composite +6 ESE which provides interaction for hnRNPQ1 [109]; and (ii) the AG-rich ESE that provides overlapping binding sites for the splicing factors PSF [102], hnRNPM [103] and hTra2-β1 [113]. The direct interactions of hTra2-β1 with the alternative splicing factors SRp30c [114], hnRNPG and RBMX/Y [115] increase the splicing activity of ESE-bound hTra2-β1, stimulating in turn exon-7 inclusion. Interestingly, a silent C/G transition identified in AG-rich ESE at position +27 (codon Gly287) in some SMA II or III patients which present mild clinical phenotypes, creates an ESE for SRSF1, which in turn promotes exon-7 splicing and the production of full-length *SMN2* mRNAs [116]. However, this transition also disrupts a splicing-inhibitory hnRNPA1 binding site indirectly promoting *SMN2* exon-7 inclusion [117].

SRSF1 [98], polypyrimidine tract-binding protein-associated splicing factor (PSF) [102] and hnRNPM [103] interact with exonic splice enhancer (ESE) sequences that promote the inclusion of exon-7 in *SMN1*, while hnRNPM further stimulates the recruitment of the splicing factor U2AF65 to the flanking intron-7 [103] (Figure 4**A**). In contrast, several base changes in *SMN2* exon-7 and flanking introns inhibit exon-7 inclusion. They alter the +6 ESE sequence leading to reduced interaction with SRSF1 [98,^104^,105], and form composite exonic splice silencer (ESS) sequences that bind splicing inhibitors hnRNPA1 [106,107] and Sam68 [108], as well as intronic splice silencer (ISS) sequences that interact with a 33 kDa protein p33 [110], hnRNPA1 [112], hnRNPA2 and hnRNPB1 [111]. Inclusion of *SMN2* exon-7 is stimulated by ESE-binding of hnRNPQ1 [109], PSF [102], hnRNPM [103] and hTra2-β1 [113], together with hTra2-β1-associated alternative splicing factors Srp30c [114], hnRNPG and RNA binding motif protein chromosome X or Y (RBMX/Y) [115]. Interestingly, a mutation in SMA patients with a mild clinical phenotype provides an ESE for SRSF1 [116] while disrupting an ESS for hnRNPA1 [117] which promote in turn the splicing of exon-7. A schematic of the complex regulation of *SMN2* exon-7 splicing is provided in Figure 4**B**. Notably, SMA patients develop respiratory impairment in the late stages of disease. Hypoxia was reported to induce both hnRNPA1 and Sam68 protein levels, promoting *SMN2* exon-7 skipping and further reduction of SMN protein levels. SMA SMNΔ7 mice housed in hyperoxia conditions, in contrast, displayed increased splicing of exon-7 and improved motor function [118].

SMN proteins form part of a large oligomeric SMN complex of nine core-proteins composed of SMN (Gemin1), Gemins 2–8 and Unrip in mammals. This complex is found in the nucleus in Gemini of coiled (GEMs) bodies often associated with Cajal bodies where it is thought to play a role in RNA polymerase II dependent transcription and/or pre-mRNA splicing. The SMN complex is required in the cytoplasm for the assembly of uridine-rich small nuclear RNPs (U snRNPs) that compose the spliceosome, providing a binding-platform for the Sm core-domain and the targeted recruitment of snRNA [119,120]. The integrity of the spliceosome is indeed altered in SMA [121], and alternative splicing of several pre-mRNAs is affected in the disease [122]. SMN depletion affects snRNA stoichiometry and promotes widespread pre-mRNA splicing defects [123] altering the alternative splicing of U12-intron-containing genes, including Stasimon (*CG8408*) [124] and Neurexin2a (*Nrxn2a*) [125] which are involved in neuromuscular junction transmission and synapse assembly/synaptic transmission, respectively. The SMN complex also plays a key role in axonal mRNP assembly and transport [126] where SMN-containing granules interact with hnRNPR, which in turn binds to the 3′UTR of *β-actin* mRNA [127], and with Hu-antigen D (HuD) that interacts with the candidate plasticity gene 15 (*cpg15*) mRNA in neuronal processes [128]. Over 200 neuronal mRNAs are associated with SMN complexes, and approximately one third co-localize in axons and neurites [129]. SMN also binds methylated lysine 79 of histone 3 (H3K79), a post-translational modification marker associated with splicing, suggesting that epigenetic dysregulation may also occur in SMA [130]. In addition, mice deficient for miRNA processing in spinal motor neurons exhibit features of SMA, indicating that miRNA processing plays an essential role in the development and integrity of spinal motor neurons [131].

In SMA, reduction in SMN levels also induces down-regulation of Gemins 2/3, hnRNPQ, hTra2-β1 [132], and promotes *SMN2* exon-7 skipping [133], thereby exacerbating the SMA phenotype. In summary, SMA is primarily caused by SMN protein-loss-of function (Figure 2**A**) that results in broad splicing and axonal mRNP trafficking defects.

## ALS

ALS is a multifactorial disease caused by mutations in one of over 20 genes encoding proteins with a variety of functions [11]. Until recently, most understanding of the mechanisms of motor neuron injury emerged from the study of *SOD1* mutations in experimental models and human biosamples. In the presence of mutant superoxide dismutase 1 (SOD1) and in sporadic cases of ALS, multiple interacting factors contribute to the neurodegenerative process, including oxidative stress, excitotoxicity, mitochondrial dysfunction, disruption of the cytoskeleton/axonal transport, protein aggregation and altered glial-motor neuron cross-talk [134]. More recently, alteration of mRNA metabolism was identified as a major dysregulated pathway in most common subtypes of ALS. In 2006, the nuclear loss and aggregation of altered forms of the RNA-processing TAR DNA-binding protein 43 (TDP-43) observed in neuronal and glial cells of ALS patients focused attention on altered RNA processing [135,136]. TDP-43 proteinopathy was found to form the hallmark of most familial and sporadic ALS cases. Ubiquitinated, phosphorylated TDP-43 wild type or mutant protein and carboxyl-terminal degradation products constitute major components of intranuclear and cytoplasmic neuronal inclusions that are observed in the majority of ALS variants except for those caused by *SOD1* mutations, highlighting alteration of mRNA-processing [137] and mRNA-binding [138] as one of the critical pathophysiological disease mechanisms [10]. Interestingly, oculomotor neurons that are relatively spared during the course of neurodegeneration in ALS show a specific transcriptome profile with decreased susceptibility to excitotoxicity [139], suggesting that selective neuronal death is induced by an inability of affected neurons to cope with increased stress in relation to ALS mutations, environmental factors and/or ageing [140].

The most common genetic causes of familial ALS involve autosomal-dominant mutations in the following four genes: *C9ORF72* (uncharacterized) in 40–50% cases [89,90], also most commonly mutated in frontotemporal dementia (FTD), *SOD1* [141] in 20% [142], *TARDBP* (encoding the TDP-43 protein) [143] and *FUS* (fused in sarcoma) [144,145] in 4% [146] of fALS cases, respectively. These mutations can also be found in a varying proportion of sporadic ALS cases. Widespread dysregulation of gene expression was observed in these ALS subtypes as well as some other less common genetic variants described below.

### C9ORF72-related ALS

In contrast to TDP-43 and FUS, the C9ORF72 protein does not display homology to RNA-binding proteins and is not thought to play a direct role in mRNA metabolism. *C9ORF72* encodes a protein of uncharacterized function which might belong to the family of DENN-containing proteins, GDP-GTP exchange factors (GEFs) for Rab GTPases domain involved in the regulation of membrane trafficking [147]. ALS mutations are associated with pathological intronic hexanucleotide GGGGCC repeat expansions [89,90] containing from >30 up to several thousand repeats [148,149] in the first intron of the *C9ORF72* gene. The *C9ORF72* RNA repeat expansions form stable parallel uni and multimeric G-quadruplex structures 150–152 which are well known for avidly interacting with RNA-processing factors. Both sense 89,91,153–155 and anti-sense 152,155–157 intraneuronal RNA foci have been observed in association with the *C9ORF72* hexanucleotide expansion and form a pathological hallmark of C9ORF72-related ALS.

Three potential molecular mechanisms of gene expression alteration and neuronal injury have been proposed and may coexist simultaneously: (i) haploinsufficiency due to decreased expression from the altered *C9ORF72* allele(s) 89,158–162 (Figure 2**A**); (ii) RNA mediated gain-of-function toxicity by the GGGGCC-expanded intron that prevents aberrantly bound/sequestered RNA-processing factors from functioning normally in the nucleus 91,92,153–155,163,164; and (iii) protein gain-of-function toxicity by dipeptide repeat proteins (DPRs) aberrantly generated from RAN translation of the expanded intron-containing mRNA [156,^165^,166] (Figure 3**A**–**C**).

Haploinsufficiency was reported by several groups [158,^159^,^161^,162] and in a zebrafish model of C9ORF72-related ALS [160]. However, decreased mRNA expression was not observed in induced pluripotent stem cell (iPSC)-derived neurons [154]. Similarly, we [167] and others [161] have also reported that C9ORF72 mRNA steady-state levels are not altered in ALS cases with small GGGGCC repeat lengths. In addition, not a single mutation has been identified in the *C9ORF72* coding sequence, suggesting that pathogenicity due to loss-of-function is less likely [168]. On the other hand, a body of evidence is accumulating for RNA gain-of-function toxicity. Antisense oligonucleotides have been shown to rescue the GGGGCC-expanded *C9ORF72*-mediated RNA toxicity [153,^154^,156]. We [92] and others 91,153–155,163,164 are proposing RNA mediated gain-of-function toxicity as one contributing mechanism operating to prevent aberrantly bound/sequestered RNA-processing factors from functioning normally in the nucleus, which in turn leads to broad alteration of gene expression in C9ORF72-related ALS. On the other hand, DPRs are abundant in extra-motor areas of the CNS such as the cerebellum and account for the characteristic pathology of p62-positive, TDP-43-negative inclusions seen in the CNS of C9ORF72-related ALS patients. DPRs were recently shown to *in vitro* alter RNA biogenesis and kill cells [169], as well as causing neurodegeneration in drosophila [170]. However, DPRs seem to be most abundant in areas of the CNS outside the motor system, which is responsible for the key disease-related clinical features, raising also the possibility of a potential beneficial/neuroprotective role of DPR generation.

### *SOD1*-related ALS

*SOD1* encodes a ubiquitously expressed free radical scavenging enzyme. ALS *SOD1* mutations [141] lead to increased oxidative stress and neurodegeneration associated with mitochondrial dysfunction [140,171]. SOD1 is not thought to play a direct role in RNA metabolism. However, biomarkers of RNA oxidation are detectable in human ALS and as an early indicator of oxidative stress in mutant *Sod1* mice. The transcriptomes of dissected spinal cord motor neurons from SOD1-related ALS models [172] were further shown to be significantly altered with up- and down- regulation of over 1000 transcripts whose products are involved in various metabolic pathways, including those controlling neuronal survival and death. In particular, in motor neurons that survived the disease process in human *SOD1*-related ALS, anti-apoptotic phosphatidylinositol 3-kinase and protein kinase B (AKT3) were up-regulated with a concomitant reduction in the level of phosphatase and tensin homologue (PTEN) which inhibits the pro-survival AKT pathway, suggesting a mechanism for how these intact motor neurons survived the neurodegenerative process. Therapeutic modulation of this pathway is now being developed as a potential neuroprotective approach.

More than 170 missense mutations of *SOD1* have been reported in ALS cases (ALSoD Consortium; http://alsod.iop.kcl.ac.uk/). They are distributed over the full length of the 154 amino acids of the human SOD1 protein, suggesting that loss-of-function mutations are unlikely. Furthermore, knock down of *Sod1* does not trigger ALS in mice, and several SOD1 mutants, including A4V and G93A, retain virtually normal superoxide dismutase activity. The pathophysiological mechanisms of SOD1 mutations are thought to involve protein toxic gain-of-function (Figure 2**B**) due to protein misfolding and formation of ubiquitinated intracytoplasmic neuronal and astrocytic inclusions in the CNS of SOD1-related ALS patients and mice [173,174].

### TDP-43 and FUS-related ALS

*TARDBP* and *FUS* encode ubiquitously expressed DNA/RNA-binding proteins implicated in multiple aspects of gene expression regulation, including transcriptional control, alternative splicing of mRNA [175,176], axonal transport of mRNA [177] and biogenesis of miRNA [178,179]. More recently, TDP-43 was also shown to play additional roles in translation control [180,181]. Several thousand TDP-43 and FUS RNA-binding sites have been characterized on pre-mRNA molecules, including those involved in splicing functions of long pre-mRNAs essential to neuronal development and integrity [176,^182^,183]. Alternative splicing of pre-mRNAs was found to be broadly altered in TDP-43 and FUS-related ALS, leading to the dysregulation of normal neuronal gene expression with the synthesis of thousands of aberrantly spliced mRNA molecules 182,184–189. In particular, these recent transcriptome studies highlighted an alteration of levels and/or splicing of genes involved in RNA processing, synthesis of neurotrophic factors and synaptic function. Up to one-third of the transcriptome is altered in TDP-43 transgenic mouse models with specific alterations due to the Q331K ALS mutation [186]. Overexpression of FUS in mice also causes ALS, with progressive loss of motor neurons in an age- and dose-dependent response [190]. On the other hand, FUS interacts directly with the SMN complex [191] that associates with GEMs for the biogenesis of snRNPs. GEMs are significantly reduced in SMA, in patient fibroblasts expressing TDP-43 or FUS mutations [191] and in motor neurons of TDP-43 transgenic mice [192]. ALS-associated TDP-43 mutations increase interaction with FUS [193], and spliceosome integrity was found to be affected in TDP-43-related ALS [121]. TDP-43 interacts also with GEMs via SMN [194]. These observations suggest shared defective RNA splicing mechanisms between SMA and TDP-43/FUS related ALS [121,191].

FUS and TDP-43 are hnRNP proteins which shuttle between the nucleus and the cytoplasm. They are composed, respectively, of one and two RRM domains flanked by carboxyl-terminal arginine/glycine (RG)-rich regions, also called the prion-domain because these unstructured regions have a propensity for aggregation. FUS also exhibits an RG-rich region in its amino-terminus. The vast majority of ALS mutations cluster in the RG-rich regions in exon 6 of TDP-43 and in exons 3–6 or 12–15 of FUS. These mutations disrupt protein : protein interactions, including those of TDP-43 with the splicing hnRNPs A1, A2/B1, C1/C2 and A3 [195]. ALS mutations of TDP-43 and FUS also alter the transportin-mediated nuclear localization import, resulting in predominantly cytoplasmic mislocalization and the formation of stress granules [196,197]. It remains unknown whether motor neuron injury is caused by loss of normal nuclear functions of TDP-43/FUS (disrupting transcriptional regulation, pre-mRNA splicing, sorting to distinct cytoplasmic compartments or processing of noncoding RNAs) and/or whether additional toxic gain(s) of function, such as cytoplasmic mislocalization in soluble or aggregated forms, are responsible for disease (Figure 2**A**,**B**). However, toxicity does not seem to depend on nuclear localization of mutant TDP-43 or on the formation of intracytoplasmic inclusions, but rather on the increased cytoplasmic mislocalization of soluble ALS-linked TDP-43 mutant proteins [198,199]. Also, the alteration of the axonal mRNA transport is likely to contribute to motor neuron dysfunction in TDP-43 related ALS [200].

### Minor subtypes of ALS that exhibit RNA dysregulation

Further evidence of dysfunctional RNA metabolism has been provided by autosomal-dominant mutations in genes encoding senataxin [201] and angiogenin [202,203], two other RNA-binding proteins, involved in rare juvenile familial and adult onset forms of ALS, respectively. The DNA/RNA helicase senataxin (*SETX*) is predicted to play roles in several steps of gene expression, including functions associated with RNA processing and the maintenance of genome integrity. Angiogenin (*ANG*), the expression of which is increased during hypoxia to promote angiogenesis, is a tRNA-specific RNase that also regulates the transcription of ribosomal RNA. ALS mutations of *ANG* are likely to act through a loss of function, as overexpression extends the lifespan of ALS SOD1 mutant mice. The mechanism(s) by which mutations in *SETX* and *ANG* cause ALS remains to be determined.

Additional genetic mutations were more recently identified in elongator protein 3 (*ELP3*) [204], *TAF15* [205,206] and Ewing's sarcoma breakpoint region 1 (*EWSR1*) [207]. Products from these genes are also involved in regulating RNA metabolism. The histone H3/H4 acetyl transferase ELP3 comprises one subunit of the elongator complex that regulates transcription elongation by the RNA Polymerase IIO, post-transcriptional processing of tRNA, as well as acetylation of α-tubulin in microtubules. TAF15 and EWRS1 proteins are functionally and structurally related to FUS. They form the FET family of proteins (FUS-EWS-TAF15) and play a regulatory role in transcription and alternative splicing. Also, poly-Q expansions described in the coding sequence of Ataxin-2 (*ATXN2*), a gene product involved in the translation control of the circadian rhythm [208,209], were shown to be significant susceptibility factors associated with ALS [210]. ATXN2 localizes with stress granules and TDP-43 in neurons. Products of genes that modulate stress granules are potent modifiers of TDP-43 toxicity in *Saccharomyces cerevisiae* and *Drosophila melanogaster*, and TDP-43 interacts with the polyA-binding protein (PABP) [211]. Mutations in the RNA/DNA-binding protein Matrin 3 (MATR3) that also interact with TDP-43 were shown to cause ALS [212]. Furthermore, a mutation causing ALS was found in the prion domain like of hnRNPA1 [213]. The precise mechanisms of neurodegeneration triggered by mutations in genes encoding these various RNA-processing factors remain poorly understood.

## RNA-mediated mechanisms of neurodegeneration

Expression level alteration and/or sequestration of proteins involved in the process of gene expression are common mechanisms implicated in neurodegeneration; however, they usually diverge in the targets identified so far across the various RNA-mediated neurodegenerative diseases. Comprehensive transcriptome studies have recently been reviewed in HD [214], SCA [215], SMA [216] and ALS [9,217]. Here, Tables 1 and 2, respectively, focus on the recent transcriptome analysis of pathogenic variants in the nonmotor (HD, SCA) and motor (SMA, ALS) neurodegenerative diseases that are the focus of this review. In common, transcriptome studies of pathogenic mutations found in *HTT*, *ATXN2*, *ATXN7*, *SMN*, *TARDBP/TDP-43*, *FUS* and *C9ORF72* all suggest that most changes alter the expression of genes associated with neuronal specificity, plasticity and synaptic function. This provides, in turn, an attractive hypothesis explaining why neurons are preferentially more sensitive than other cell types to the pathogenic mutations.

**Table 1:** Recent transcriptome studies of pathogenic mutations in RNA-mediated nonmotor neuron neurodegenerative diseases (HD and SCA)

Study	Samples	Platform	Measure of differential expression (no. differentially expressed)	Main findings
HD:				
Jacobsen *et al*. [218]	RNA extracted from mouse embryonic stem cells expressing wild-type **huntingtin** with 7Q, and knock-in expansions of length 20Q, 50Q, 91Q and 111Q (*n* = 3–6 replicates per group).	Affymetrix Mouse 430A microarray	Correlation examined between expansion length and gene expression, Pearson coefficient > 0.8, *P* < 0.001 (25 probes positively correlated, 48 probes negatively correlated)	Genes correlated with expansion size distinct from genes dysregulated by knockdown of huntingtin, but are within similar functional pathways including energy and lipid metabolism.
Feyeux *et al*. [219]	RNA extracted from six embryonic stem cell and corresponding neural stem cell lines expressing **huntingtin** with 40–51Q expansions and four wild-type lines	Illumina Human WG-6 v3 microarray	Compared with wild type, Limma, *P* < 0.001 (embryonic stem cell: 163, neural stem cell: 66)	Dysregulated gene expression functionally enriched for energy and lipid metabolism and gene expression; no overlap with data sets analysing gene expression in brain tissue from symptomatic adults
Mattis *et al*. [220]	RNA extracted from neural stem cells and striatal neurons derived via induced pluripotent stem cells from patients with 60–180Q expansions of **huntingtin** and wild-type controls (*n* = 2–3 each group)	Human Affymetrix Exon 1.0 ST microarray.	Comparison to wild type, anova, FC > 2 (NSC: 1601; striatal neurons: not stated)	Neural stem cells: Dysregulated genes functionally enriched for signalling, cell cycle, axonal guidance and neural development. Some changes specific to longer or shorter repeat length. Striatal neurons: Dysregulated genes in striatal neurons functionally enriched for proliferation, signalling and cellular assembly
Soldati *et al*. [221]	RNA extracted from striatal neurons obtained and grown from mice expressing two **huntingtin** alleles with 109Q expansions, nonexpanded 7Q alleles.	Illumina MouseWG-6 v2.0 microarray	Compared with 7Q homozygous cells: Benjamini Hochberg, FDR < 0.02 (1013↑ and 1342↓)	Dysregulated genes functionally enriched for nervous system development and function. Down-regulated genes in these functional categories were enriched for REST binding sites.
Lewandowski *et al*. [222]	RNA extracted from posterior and anterior caudate, and S1 of patient (mean **huntingtin** expansion 41Q) and control brains (*n* = 10 each group)	Affymetrix Human U133A 2.0 microarray	Compared with control, repeat measures anova, controlling for region, *P* < 0.05 (3)	Focused on down-regulation of PPP1R7 which is implicated in neuronal function
Lee *et al*. [223]	RNA extracted from lymphoblastoid cell lines derived from 107 patients with **huntingtin** expansions of 15–92Q	Affymetrix Human U133A 2.0 microarray	Pearson correlation between probe expression and expansion length used to derive a biomarker of expansion length	Correlated genes involved in ribosomal function, transcription, nucleic acid metabolism, adhesion, energy metabolism, hormone response, synaptic transmission, neurological process
SCA7				
Chou *et al*. [224]	RNA extracted from the cerebellum of symptomatic transgenic mice expressing human **ataxin 7** with a 52Q expansion, and wild-type mice (*n* = 4 each group)	Affymetrix Mouse 430A microarray	Compared with wild type, *t*-test, *P* < 0.05, FC > 1.4 (10↑ and 34↓)	Down-regulated genes involved in neuronal function, protein processing, heat shock response and glial function. Up-regulated genes include RNA binding proteins.
Friedrich *et al*. [225]	RNA extracted from laser-captured Purkinje neurons from cerebellum of transgenic mice expressing human **ataxin 7** with 90Q expansion and wild type mice	Affymetrix Mouse 430A microarray	P7E: Compared with wild type, FDR *P* < 0.2 (13)	Down-regulated genes involved in vulnerability to excitotoxicity.
SCA17				
Ren *et al*. [226]	RNA extracted from heads of transgenic flies expressing human **TBP** with a 34Q or 80Q expansion, at days 5, 28 and 35. Day 28 is immediately prior to the onset of motor symptoms in the 80Q flies.	Affymetrix Drosophila Genome 2.0 microarray	Compared with 34Q samples at each time point, *t*-test, *P* < 0.05; FC > 1.4 (536)	Differentially expressed genes functionally enriched for pathways, including oxidation and mitochondrial function. Pearson correlation analysis implicated transcription factor Su(H) in control of dysregulated gene expression.
SCA2				
Damrath *et al*. [227]	RNA extracted from cerebellum, brainstem and liver of transgenic mice expressing **ataxin 2** with a 42Q expansion at 6 months (presymptomatic) and 18 months (symptomatic), and wild-type mice (*n* = 3–4 per group)	Affymetrix Mouse 430A microarray	Compared with wild type, Benjamini Hochberg (6 months: no significant gene expression changes. 18 months: cerebellum 20 genes; brainstem 14 genes, liver 30 genes)	In cerebellum, identified dysregulation of Adam1a and Fbxw8 which are neighbouring genes of ataxin 2. Fbxw8 implicated in neuronal dendrite formation
SCA3				
Hsieh *et al*. [228]	RNA extracted from SK-N-SH cells engineered to stably express **ataxin 3** with 26Q or 78Q expansion (*n* = 2 each group)	ABC Human UniversoChip 8k-1 microarray	Comparison with 26Q expressing cells, genes ranked by fold change	Most differentially expressed gene by fold change was CA11; function of CA11 unknown.
SCA28				
Mancini *et al*. [229]	RNA extracted from lymophoblastoid cell lines derived from patients with mutations of **AFG3L2** or matched controls (*n* = 4–6 each group)	Affymetrix Human U133A 2.0 microarray	Compared with controls, rank product, FDR < 0.005 (35↑ and 31↓)	Differentially expressed genes implicated in regulation of cell proliferation, cell death, cell adhesion, oxidative stress and chemical homeostasis

FC, fold change; FDR, false discovery rate; HD, Huntington's disease; SCA, spinocerebellar ataxia.

**Table 2:** Recent transcriptome studies of pathogenic mutations in RNA-mediated neurodegenerative diseases of the motor system (SMA and ALS)

Study	Samples	Platform	Measure of differential expression (no. differentially expressed)	Main findings
SMA				
Garcia *et al*. [230]	Smn-null and wild type drosophila lavae (*n* = 2 each group). RNA extracted when Smn-null lavae first display motor phenotype	Illumina HiSeq 2000	Gene level: Compared with wild type; Fisher's exact test. Exon level: Compared with wild type, DEXSeq, *P* < 0.05 (2153 splicing changes); MISO, Bayes factor > 100 (2484 splicing changes)	Comparison of gene level analysis with modENODE data [231] consistent with developmental arrest in the Smn-null animals; splicing changes exceeded normal developmental changes
Zhang *et al*. [232]	Microdissected motor neurons and glial cells from spinal cord of SMN-deficient and wild-type mice at postnatal day 1 before MN pathology develops (*n* = 2 each group)	Illumina HiSeq 2000	Compared with wild type. Gene level:, DESeq, *P* < 0.05, FC > 2 (Motor neurons: 138↑ and 110↓; Glial cells: 125↑ and 87↓). Exon level: MISO, Bayes factor > 10 (Motor neurons: 104; glial cells: 86 splicing changes)	Dysregulation of genes implicated in synaptogenesis. Minimal overlap of transcriptome changes between cell types despite broadly similar genes expressed
See *et al*. [125]	Zebrafish embryos at 48h post-fertilization injected morphilino *vs.* SMN, Gemin2 or scrambled (*n* = 6–7 each group)	Custom zebrafish microarray [233]	Compared with scrambled, *t*-test, *P* < 0.05, fold change > 2 (1545 transcripts)	Down-regulation of Neurexin2a which is involved in synaptic function. Knockdown of Neurexin2a phenocopied the SMA model in zebrafish
Acsadi *et al*. [234]	NSC34 cells transfected with shRNA *vs.* SMN or scrambled	StellARray™ microarray NE0100-MM96	NSC34 cells, *vs.* scrambled fold change > 2 (5 genes). Note that custom array contains only genes linked to neurodegeneration.	SNCA down-regulation highest fold change. Change confirmed in peripheral and CNS tissue from SMA patients. SNCA is implicated in synaptic function and PD.
C9orf72-linked ALS				
Donnelly *et al*. [153]	RNA extracted from motor cortex, fibroblasts and iPSNs from C9orf72+ ALS patients and controls (*n* = 2–4 each group). Followed by transfection of C9orf72+ iPSNs with ASO *vs.* C9orf72	Human Affymetrix 1.0 ST Exon microarray.	Compared with control, *t*-test *P* < 0.05. (Fibroblasts: 487 genes↑ and 1238 genes↓; iPSNs: 984 genes↑ and 2314 genes↓; motor cortex: 1870 genes↑ and 5114 genes↓)	Significant number of differentially expressed genes in common between C9orf72+ iPSNs and C9orf72+ motor cortex. ASO treatment normalized gene expression in C9orf72+ iPSNs.
Sareen *et al*. [154]	RNA extracted from fibroblasts and iPSNs from C9orf72+ ALS patients and controls (*n* = 4 each group). Followed by transfection of C9orf72+ iPSNs with ASO *vs.* C9orf72 or scrambled ASO	Illumina HiSeq.	Compared with control, *t*-test *P* < 0.05	Differentially expressed genes in iPSNs functionally enriched for synaptic transmission. ASO *vs.* C9orf72 partially corrected gene expression in C9orf72+ iPSNs.
Lagier-Tourenne *et al*. [155]	RNA extracted from fibroblasts of C9orf72+ ALS patients, non-C9orf72 ALS patients and controls (*n* = 4 each group). Followed by transfection of C9orf72+ samples with ASO *vs.* C9orf72	Illumina Hi-Seq	C9orf72+ compared with controls, FDR < 0.05 (122 genes↑, 34 genes↓)	Treatment with ASO *vs.* C9orf72 failed to reverse expression signature of C9orf72+ fibroblasts.
Ismail *et al*. [235]	RNA extracted from lymphoblastoid cell lines derived from C9orf72+ ALS patients, non-C9orf72 ALS patients and controls (*n* = 10–16 each group)	Human Affymetrix Genome U133 Plus 2.0 microarray	Compared with controls. PPLR [236], *P*LikeValue < 0.05 (C9orf72+ *vs.* control: 319 genes; C9orf72+ *vs.* non-C9orf72: 294 genes)	Differentially expressed genes functionally enriched for NF-κB activity. Top differentially expressed gene CXCL10 down-regulation in C9orf72+ samples; this gene is controlled by NF-κB and is a positive prognostic marker in sporadic ALS [237].
TDP-43-liked ALS				
Huang *et al*. [188]	Astrocytes purified from (GFAP)-tTa/TRE-TDP-43^M337v^ double transgenic rats. RNA extracted after 3, 4 or 6 days of induction of mtTDP-43 expression	Microarray	Compared with baseline FC > 2 at ≥1 time point (449 genes) and progressive change in FC over induction time points	Induction of TDP-43^M337V^ expression altered expression of secreted factors; in particular, expression of neurotrophic factors increased and expression of neurotoxic factors decreased.
Arnold *et al*. [186]	Transgenic mice produced which express TDP-43^Q331K^, or TDP-43^wild-type^ throughout CNS. RNA was extracted from cortices and spinal cords of 2-month-old mice and compared with nontransgenic mice.	Affymetrix ‘A = chip’ microarray.	Compared with nontransgenic. Absolute separation score < 0.3, *q* < 0.05 (Cortex: TDP-43^wild-type^ 824 exons alternatively spliced; TDP-43^Q331K^ 1195 exons; Spinal cord: TDP-43^wild-type^ 4462 exons, TDP-43^Q331K^ 4399 exons)	Differentially spliced exons in mutant, but not wild-type transgenic mice enriched for known TDP-43 binding sites. Differentially spliced exons in TDP-43^Q331K^ which overlapped between cortex and spinal cord enriched for synaptic function
Highley *et al*. [189]	RNA extracted from fibroblasts derived from mtTDP-43 ALS patients, mtSOD1 ALS patients, sporadic ALS patients and controls (*n* = 3–6 each group)	Human Affymetrix Exon 1.0 ST microarray	Compared with controls, ancova *P* < 0.01 (mtTDP-43: 1070 genes↑ and 433 genes↓, 12 280 exons; mtSOD1: 6335 exons; sporadic ALS: 1140 exons)	Functional enrichment in mtTDP-43 differentially expressed/spliced genes for categories related to RNA processing. Alternative splicing much more abundant in mt-TDP-43 *vs.* other ALS patients

ALS, amyotrophic lateral sclerosis; ASO, antisense oligonucleotide; FC, fold change; FDR, false discovery rate; iPSNs, induced pluripotent stem cell-derived motor neurons; SMA, spinal muscular atrophy.

However, it still remains unclear whether widespread gene expression changes are the result of an exclusive alteration of mRNA biogenesis/processing, an indirect effect due to DNA/RNA/protein damage via oxidative stress/mitochondrial dysfunction, or a combination of these exhibiting in turn both loss-of-function effects and toxic gain-of functions via the formation of RNA foci and/or protein aggregates. The apparent lack of convergence in the altered RNA/protein targets does not necessarily imply that there are not common mechanisms involved. The large number of dysregulated gene expression events identified so far and the involvement of redundant pathways, either up- or down-regulated, complicate the interpretation of observations. For example, energy/lipid pathways are similarly dysregulated by either poly-Q HTT expansions or *HTT* knockdown, but the particular genes whose expression is altered are different [218]. Also, TDP-43 and FUS were shown to bind distinct RNA sites, but their functions overlap in the alternative splicing of pre-mRNAs with long introns, many of which encode genes associated with neuronal integrity [176,^182^,183].

Expanded pre/mRNA in repeat disorders were reported to form RNA foci and avidly bind/sequester splicing factors such as SRSF1 in SCA31/C9ORF72-related ALS [85,^91^,92], SRSF2 in SCA36/C9ORF72-related ALS [88,^91^,92] and MBNL1 in HD/SCA8 [46,96]. Disruption of GEMs is observed in both SMA and TDP-43/FUS-related ALS [191,192], and concordantly, the integrity of spliceosomes was found to be altered in both diseases [121]. Expression level alteration/sequestration of RNA-processing factors such as RRM-containing splicing factors (hnRNPs, SRSFs) further leads to large splicing alterations in SMA 123–125, TDP-43/FUS-related ALS 182,184–189 and C9ORF72-related ALS [153,154].

RAN translation has been observed in SCA8 [97] and C9ORF72-related ALS [156,^165^,166]. However, how repeat-expanded pre-mRNAs are exported into the cytoplasm remains unknown. Increased binding of mRNA export adaptors (ALYREF, SRSF1, SRSF3, SRSF7) on GGGGCC-expanded C9ORF72 pre-mRNAs [92] may override normal nuclear retention and inappropriately license repeat-expanded pre-mRNAs for nuclear export [28,238], thus allowing RAN translation to occur. On the other hand, repeated sequence expansions such as those observed in HD, SCA and C9ORF72-related ALS are likely to generate transcriptional stress with the formation of R-loops that are more susceptible to DNA damage and may result in increased genome instability (for a recent review, see Skourti-Stathaki & Proudfoot [239]). Interestingly, the ALYREF/THOC4 (THO complex subunit 4) subunit of the TREX complex that links transcription elongation to mRNA nuclear export and genome stability was found to be sequestered by pathogenic C9ORF72 RNA hexanucleotide repeat expansions [92]. Dysregulation of miRNA biogenesis was also reported to play an important role in HD [48,^53^,56], in SMA [131] and in FUS-related ALS [240].

Whether RNA-mediated neurodegeneration and its various clinical presentations are driven by distinct mechanisms specific to each affected type of neuron or by a common pathophysiological core of dysregulated targets remains to be determined.

## Current challenges: identifying altered levels/sequences of RNA molecules and proteins causing neurodegeneration

As described earlier, RNA-mediated neurodegenerative diseases exhibit widespread dysregulation of gene expression with alteration of most RNA classes (mRNA, miRNA, rRNA and tRNA) and disruption of multiple RNA-processing steps. Accordingly, transcriptome studies have revealed dysregulation of hundreds to thousands of RNA molecules in the presence of disease-causing mutations [217].

Transcriptomics as a research methodology has contributed greatly to our understanding of the mechanisms of neuronal cell death [241]. However, there are clear limitations inherent within traditional transcriptomics studies due to both the measurement of steady-state levels and the nature of available samples from which RNA is obtained (i.e. cell/animal models and *post mortem* tissues enriched for neurons that survived the neurodegenerative process) [242,243]. Identifying events causing neurodegeneration is also challenging because of the widespread and varied components of RNA dysregulation. For example, the large number of reported splicing anomalies constitutes a major hurdle in separating causal molecular events leading to neurodegeneration from those that are downstream consequences of the initial perturbations of gene expression. Another problem lies in the fact that very often, the expression level of a given mRNA transcript is not necessarily representative of the level of its corresponding protein. Counter-intuitively, an increase in the steady-state level of a particular mRNA is often associated with a decrease in the corresponding protein level as the cell tries to counteract the down-regulation of the protein by increasing synthesis/stability of the mRNA [244]. Practically, it is also difficult for researchers to determine which altered RNAs should be considered more or less important in a given biological context. It would be tempting to assume that the RNAs with the greatest relative fold changes would have the highest chance of being pathophysiologically relevant but, as we have seen with the previous example, a significant increase in mRNA level could be misleading, particularly as in most cases, it is the protein product that is more likely to influence cellular health. Additional complexity comes from the fact that intronic repeat sequences such as GGGGCC expansions in *C9ORF72* can be translated into toxic proteins [156,^165^,^166^,^169^,^170^,245] which further extends the repertoire of altered gene expression that can be implicated in neurodegeneration. Furthermore, biomarkers of RNA oxidation are also detectable in human CNS samples from cases with neurodegenerative disorders. Oxidation of RNA molecules triggers a reduction in translation and simultaneously increases translational errors potentially leading to the synthesis of aberrant proteins and mitochondrial dysfunction [140,246]. Lastly, adenosine to inosine editing of miRNA and mRNA molecules by adenosine deaminase act on RNA (ADAR) enzymes is broadly affected during neurodegeneration (ALS, HD, PD, AD), leading in particular to regulatory and translational alterations of edited mRNAs [247]. Like many of the aforementioned changes, these alterations in RNA sequence are difficult to predict in advance and would therefore be unlikely to be accounted for in traditional microarray-based transcriptomics experiments, meaning that ultimately these important changes would be missed.

## Concluding remarks and future directions

As yet, the functional consequences of RNA dysregulation for the processes that trigger age-related and selective progression of neuronal cell death remain very poorly understood. The levels, frequency and identity of aberrantly spliced mRNA isoforms that undergo nuclear export and are subsequently translated into aberrant proteins are still largely uncharacterized. These aberrant proteins, many of which are likely to have essential enzymatic or structural activities that impinge upon a multitude of cellular pathways, may have either lost their wild-type biological function or will have acquired new deleterious functions [180]. This complicates the interpretation of dysregulation events as aberrant proteins may in turn trigger cascades of further dysregulation. Given that proteins are ultimately involved in controlling cell fate, identifying altered cytoplasmic levels/sequences of mRNA and the corresponding proteome is becoming crucial for understanding the molecular causes of neurodegeneration. These approaches to OMICS studies therefore represent an essential step in the development of novel neuroprotective therapeutic strategies.

Reliable subcellular fractionation of cytoplasmic RNA, as well as purification of ribosomes for the extraction and next-generation sequencing of actively translating mRNA (i.e. the ‘translatome’) will become essential methodologies that will enable dissection of the molecular events that contribute to neurodegeneration. We anticipate that emerging methodologies investigating gene expression dysregulation at the level of active protein synthesis rather than at RNA/protein steady-state levels will yield biological results of higher relevance to the understanding of disease pathophysiology. Also, the specific sequences of mRNAs undergoing translation would provide rich information on the nature of mutations and/or aberrant splicing variations that are acquired during mRNA processing, and then subsequently translated into aberrant protein expression. Recent advances in molecular biology systems have also allowed the engineering of stable inducible neuronal cell models of neurodegeneration. In these models, cellular insults, such as disease-mutated protein or expanded nucleotide repeat expression, can be turned on as required. Such systems will allow investigators to observe early events in gene expression dysregulation that are more likely to be an upstream cause rather than a consequence of disease. Furthermore, these systems allow for the tracking of the progression of this dysregulation at multiple time points, meaning that it may be possible to define the upstream pathways in the pathophysiology of neuronal injury. Significantly, these types of analysis are prohibitively difficult to perform in animal models and impossible in human *post mortem* CNS tissue. Single-cell next-generation RNA sequencing [248] on neurons derived from induced pluripotent stem cells (iPScs) produced from patient fibroblasts (which bear the exact genetic makeup that caused disease in an individual) also holds great promise for investigating the functional consequences of RNA dysregulation in primary cells that are directly relevant to the disease being studied.

Widespread dysregulation of RNA metabolism is now clearly recognized as a pathophysiological component triggering neurodegeneration in the neurodegenerative diseases that are the focus of this review. It is very likely that other neurodegenerative diseases involve widespread dysregulation of gene expression. For example, excessive phosphorylation of the ribosomal RPS15 subunit by the PD mutated LRRK2 G2019S kinase was recently shown to trigger a toxic burst in global protein synthesis [249].
